# Carbohydrate Electrolyte Solutions Enhance Endurance Capacity in Active Females

**DOI:** 10.3390/nu7053739

**Published:** 2015-05-15

**Authors:** Feng-Hua Sun, Stephen Heung-Sang Wong, Shi-Hui Chen, Tsz-Chun Poon

**Affiliations:** 1 Department of Health and Physical Education, Hong Kong Institute of Education, Rm D4-2/F-13, 10 Lo Ping Road, Tai Po, Hong Kong, 00852, China; E-Mails: fhsun@ied.edu.hk (F.-H.S.); shchen@ied.edu.hk (S.-H.C.); 2 Department of Sports Science and Physical Education, Chinese University of Hong Kong, G08, Kwok Sports Building, Chinese University of Hong Kong, Shatin, Hong Kong, 00852, China; E-Mail: ericpoontc@gmail.com

**Keywords:** running, exercise to exhaustion, follicular phase

## Abstract

The purpose of the present study was to investigate the effects of supplementation with a carbohydrate-electrolyte solution (CES) in active females during a prolonged session of submaximal running to exhaustion. Eight healthy active females volunteered to perform a session of open-ended running to exhaustion at 70% of their maximal oxygen consumption on a treadmill during the follicular phase of their menstrual cycle on two occasions. During each run, the subjects consumed either 3mL·kg^−1^ body mass of a 6% CES or a placebo drink (PL) every 20 min during exercise. The trials were administered in a randomized double-blind, cross-over design. During the run, the subjects ingested similar volumes of fluid in two trials (CES: 644 ± 75 mL *vs.* PL: 593 ± 66 mL, *p* > 0.05). The time to exhaustion was 16% longer during the CES trial (106.2 ± 9.4 min) than during the PL trial (91.6 ± 5.9 min) (*p* < 0.05). At 45 min during exercise, the plasma glucose concentration in the CES trial was higher than that in PL trial. No differences were observed in the plasma lactate level, respiratory exchange ratio, heart rate, perceived rate of exertion, sensation of thirst, or abdominal discomfort between the two trials (*p* > 0.05). The results of the present study confirm that CES supplementation improves the moderate intensity endurance capacity of active females during the follicular phases of the menstrual cycle. However, the exogenous oxidation of carbohydrate does not seem to explain the improved capacity after CES supplementation.

## 1. Introduction

The successful completion of an exhaustive endurance exercise depends on numerous factors. The availability of substrate provision, muscle glycogen storage, and hydration status are among the most commonly acknowledged factors that influence fatigue [1,2]. Fatigue during prolonged submaximal exercise at a moderate to high intensity equivalent to 65% to 85% of the maximal oxygen consumption (VO_2max_) is to a large extent the result of the depletion of muscle glycogen in skeletal muscle, and a reduction in the blood glucose concentration [3,4]. Therefore, in recent years the potential role played by the ingestion of carbohydrates (CHO) during exercise has been extensively investigated, and recommendations for CHO ingestion during endurance exercise have been made [5].

Several systematic reviews have recently summarized the effects of CHO ingestion during exercise on endurance performance [6,7,8]. These reviews have concluded that CHO ingestion plays a positive role during endurance exercise, and the potential mechanisms involved have been discussed. However, previous studies show a strong gender bias in that trained males were usually recruited as the participants [6]. Almost all of the research findings from male participants have been indiscriminately generalized and applied to female athletes. Relatively little attention has been directed toward gender differences in the effects of CHO ingestion during exercise. To our knowledge, only three studies have exclusively recruited female participants [9,10,11]. In general, female oxidize more lipids, fewer proteins, and fewer total CHO than male during endurance exercise [12]. The lower use of glycogen in skeletal muscle and lower production of hepatic glucose have also been found in female than in male [12]. Consequently, it is possible that CHO supplementation during exercise may have different effects in female than in male. There is thus an overwhelming need for more well-controlled experimental studies in female.

Although the form of CHO (liquid, semi-liquid, or solid) is not regarded to be very important when considering the potential ergogenic effects of CHO ingestion during exercise [13,14], CHO beverages are usually used. This may be mainly due to issues of hydration. Although the depletion of CHO reserves is believed to be the primary cause of fatigue in prolonged exhaustive exercise, other concurrent factors exist to limit human performance. One of the physiological perturbations that cause early fatigue is dehydration [2,15]. Fluid consumption throughout prolonged exercise has been shown to decrease dehydration and attenuate its associated effects on thermoregulation, cardiovascular functions, and exercise performance [16]. Because the ingestion of plain water may decrease plasma osmolality and sodium concentration, so as to stimulate the production of urine and reduce the urge to drink [17], it may not be appropriate for consumption during endurance exercise. A small amount of electrolytes added to the beverage could improve its palatability and encourage sufficient fluid replacement. As recommended by the American College of Sports Medicine, and supported by thoroughly investigated research findings, the regular ingestion of 150 to 250 mL of CHO-electrolyte solution (CES) every 15 to 20 min during moderate intensity exercise is an appropriate choice [2].

Therefore, the purpose of the current study was to investigate the influence of CES ingestion during prolonged submaximal running on the endurance capacity of recreationally active females.

## 2. Experimental Section

### 2.1. Subjects

Eight healthy, non-smoking, recreationally active female subjects were recruited from the university population and athletics clubs in Hong Kong. Their age, height, weight, percentage of body fat, and VO_2max_ (mean ± SEM) were 28.3 ± 1.5 years, 155.2 ± 1.4 cm, 47.8 ± 0.7 kg, 16.2% ± 0.8%, and 48.3 ± 2.1 mL·kg^−1^·min^−1^, respectively. Each subject participated regularly in various forms of endurance training (at least three sessions per week with more than 30 min in each session) and was considered recreationally active. A statement of written informed consent was obtained after the nature of the experimental procedures and the potential risks and benefits were thoroughly explained. The subjects also completed questionnaires about their medical histories and general habits. None of the subjects had an adverse medical history, major muscular condition or injury that would impede moderate intensity endurance running. In addition, the successful completion of at least one hour of endurance running at 70% of VO_2max_ was a minimum requirement for inclusion in the investigation. The procedure was approved by the Ethics Committee of the Chinese University of Hong Kong.

### 2.2. Preliminary Measurements

Two preliminary tests, the VO_2max_ test and the VO_2_-Speed test, were conducted before the two main trials were undertaken. The VO_2max_ was determined for each subject by means of a continuous, incremental, graded uphill treadmill running test (Quinton, Model 24–72) to volitional exhaustion, as described elsewhere [18]. VO_2max_ was reached when the following criteria were met: (a) a plateau of VO_2_ with increasing work rate; (b) a respiratory exchange ratio (RER) of greater than 1.15; and (c) a heart rate (HR) within 5 beats/min of the age-predicted maximal HR. The relationship between VO_2_ and submaximal running speed on a level treadmill for each subject was determined in a 16-min incremental submaximal running test. Four speeds were chosen with reference to each subject’s training status and set between 60% and 70% of VO_2max_. The subjects ran for 4 min at each speed. Expired air samples were collected using the Douglas bag method during the last minute of each 4 min period and analyzed. Each subject’s HR and rate of perceived exertion (RPE) were also monitored and recorded throughout the run. Running speeds equivalent to 70% of each individual’s VO_2max_ were determined from the results of these two tests.

One week before the first main trial, a 60-min familiarization treadmill run was also conducted to verify, and if necessary adjust, the running speed for the main trials. During this run, all of the procedures were standardized and were identical to those used during the main trials. This process enabled the subjects to be fully familiarized with all of the procedures performed and the measurements made during the main trials.

### 2.3. Experimental Procedures

Baseline analyses of the nutritional content of each subject’s normal diet were obtained on the basis of their 3-day weighed food record diaries before the main experimental trials. The amount, weight and frequency of all food and fluid consumed, and the ingestion of any extra vitamin or mineral supplements were recorded each day. The dietary records were analyzed with computer software (Food Processor 10.5, ESHA, Salem, Oregon). The subjects were instructed to repeat the same diet during the 3 days before each subsequent trial. The subjects were asked to maintain their current level of training throughout the study and to incorporate the experimental test into their training schedule as a “hard work out”. Two days before each test, the subjects were required to refrain from strenuous exercise to exclude any residual effects of fatigue from prior exercise on the experimental treatments. The subjects were also asked to avoid any foods or beverages that might induce diuresis during the 24-h period before the experiments. To increase the likelihood of euhydration before each testing session, the subjects were instructed to ingest approximately 500 ml of water in the evening before the tests.

Two open-ended runs to exhaustion at 70% of VO_2max_ were completed on a level treadmill approximately 1 month apart during the follicular phase of the participants’ menstrual cycle. The experiments were conducted on the day during which the menstrual bleeding period ended because the levels of both estradiol and progesterone were likely to be low. On each occasion, the subjects were required to consume either a diluted CES (19 mEq Na^+^ and 6% CHO, CES) or a placebo (glucose- and electrolyte-free artificially sweetened drink, PL) at every 20 min (3 mL·kg^−1^ body mass, BM). The experimental drinks were similar in color, texture, taste, and temperature. The study was conducted in the Exercise Physiology Laboratory under similar neutral environmental conditions (CES *vs.* PL: temperature, 20.3 °C ± 0.7 °C *vs.* 21.3 °C ± 0.7 °C; relative humidity, 60.6% ± 1.2% *vs.* 64.4% ± 2.3%). The experiments were administered in a double-blind cross-over design in a random order.

On the day of the experiment, the subjects reported to the laboratory after an overnight fast of at least 10 h. On arrival, the subjects were required to rest for about 15 min and drink 250 mL of water. The subject’s nude body weight was measured before and after each run. A HR monitor (Sports Tester PE3000, Polar Electro, Finland) was attached to each subject to monitor the HR during the treadmill test. Expired gases were collected for 5 min before each run. The HR, RPE, score of the perceived thirst scale (PTS), and score of the perceived abdominal discomfort scale (PAS) were also recorded. A 10-point visual analog scale was used for the PTS and PAS. A score of 1 indicated “Not Thirsty” and “No discomfort” respectively, whereas a score of 10 indicated “Very Very Thirsty” and “Very Very discomfort” respectively. After the standing gas collection, the pre-exercise capillary blood samples were collected to measure the levels of hemoglobin (Reflotron^®^ System, Boehringer Mannheim, Germany), hematocrit (Clay Adams, Autocrit Ultra 3, Englewood, NJ, USA), blood glucose (Model 1502, YSI, Yellow Springs, OH, USA), blood lactate (Model 1502, YSI), and osmolality (Vapor Pressure Osmometer 5520, Wescor Inc., Logan, UT, USA). Further capillary samples were taken during exercise to measure the concentrations of blood glucose and lactate. At the end of the exercise, the capillary blood samples were obtained again.

After the collection of the baseline measurements, a 5-min standardized warm-up began at a running speed corresponding to 60% of VO_2max_. The expired gas was collected during the last minute, and the HR, RPE, PTS, and PAS were recorded. The treadmill speed was then adjusted to a pace equivalent to 70% of VO_2max_ following the warm-up. During both trials, the subjects were required to run for as long as possible. Their endurance capacity was measured as the exercise time to volitional fatigue. Volitional fatigue was defined as the point at which the subject could no longer maintain the required running speed. To ensure maximal effort during each trial, the subjects were given strong verbal encouragement throughout the run; this encouragement was given only by blinded experimenters who were unaware of which treatment had been administered. No external time clues (*i.e.*, clocks or radio) were provided, so the participants were not aware of their performance time until every experimental test had been concluded.

The expired air samples were collected over 2 min at 15-min intervals and during the last min before perceived exhaustion during the trials. The oxygen (O_2_) and carbon dioxide (CO_2_) content were measured with a paramagnetic O_2_ analyzer and a CO_2_ analyzer (MOXUS modular metabolic system, AEI Technologies Inc., Pittsburgh, PA, USA). Both analyzers were calibrated against a “gold standard” reference gas immediately before each series of gas analyses. The VO_2_ and VCO_2_ were determined from the gas analyses, and the RER was calculated. The rates of CHO and fat oxidation were calculated from VO_2_ and VCO_2_ values using stoichiometric equations [19]. The method of collection and analysis of expired air samples has been previously described [20]. The RPE, PTS, and PAS were also recorded every 15 min and during the last minute before perceived exhaustion during the experimental trials.

### 2.4. Statistical Analysis

The data analysis was performed with SPSS software (version 16.0). A two-way analysis of variance (ANOVA) for repeated measures (Trial × Time) was used to analyze the changes in the CHO oxidation rate, RER, concentrations of blood glucose and lactate, HR, and some subjective measures, such as RPE, PTS, and PAS. Significant differences between means were identified using the Tukey *post hoc* test. A paired *t*-test was used to analyze the differences in the nutritional data, pre-exercise BM, fluid ingestion, relative exercise intensity, and time to exhaustion between the two trials. The level of significance (*p* value) was accepted at 0.05. The results are reported as mean ± SEM.

## 3. Results

No significant differences were found in the habitual dietary intake between the subjects in the CES and PL trials (Energy: 1736 ± 172 *vs.* 1582 ± 172 Kcal; CHO: 52.9% ± 3.5% *vs.* 54.7% ± 3.1%; Protein: 20.0% ± 1.9% *vs.* 19.5% ± 0.6%; Fat: 34.0% ± 3.9% *vs.* 35.5% ± 3.3%, *p* > 0.05). The subject’s pre-exercise BM was also similar between the two trials (CES *vs.* PL: 47.8 ± 0.8 *vs.* 47.9 ± 0.7 kg, *p* > 0.05). Over the course of the run, the subjects drank a total volume of 644 ± 75 ml of fluid during the CES trial and 593 ± 66 mL in the PL trial (*p* > 0.05). The participants ingested a total of 38.6 ± 4.5 g of CHO during the CES trial.

### 3.1. Exercise Time to Exhaustion

The relative exercise intensities were similar on both occasions (CES *vs.* PL: 71% ± 2% *vs.* 69% ± 2% of VO_2max_, *p* > 0.05). The exercise time to exhaustion was significantly longer (by approximately 16%) during the CES trial than during the placebo trial (CES *vs.* PL: 106.2 ± 9.4 *vs.* 91.6 ± 5.9 min, *p* < 0.05). 

### 3.2. Expired Air Analysis

**Figure 1:**
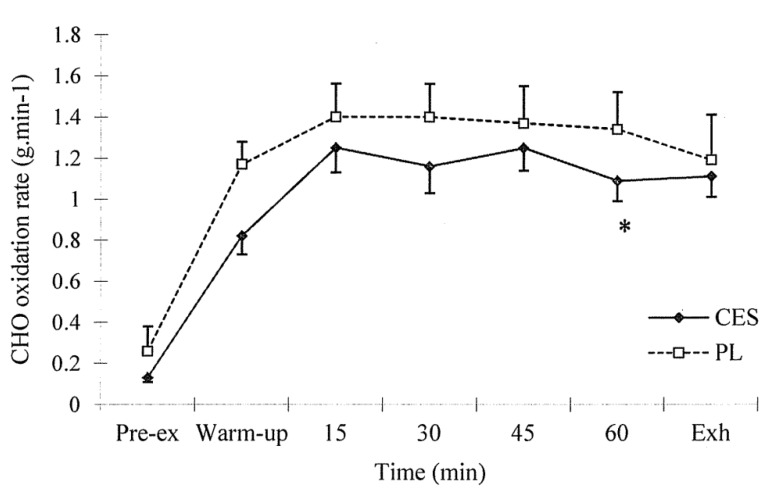
CHO oxidation rate (g·min^−1^) during treadmill running. Values (mean ± SEM) are for placebo (PL) and carbohydrate-electrolyte solution (CES) trials. * CES *vs.* PL, *p* < 0.05.

The pattern of change in the RER was similar in both trials (*p* = 0.385). The overall CHO oxidation rates during exercise were also similar in both trials (CES *vs.* PL: 1.2 ± 0.1 g·min^−1^ *vs.* 1.4 ± 0.1 g·min^−1^, *p* = 0.116). Only a transient decrease in the CHO oxidation rate was found in the 60^th^ minute of the CES trial (Figure 1).

### 3.3. Blood Sampling Analysis

**Figure 2:**
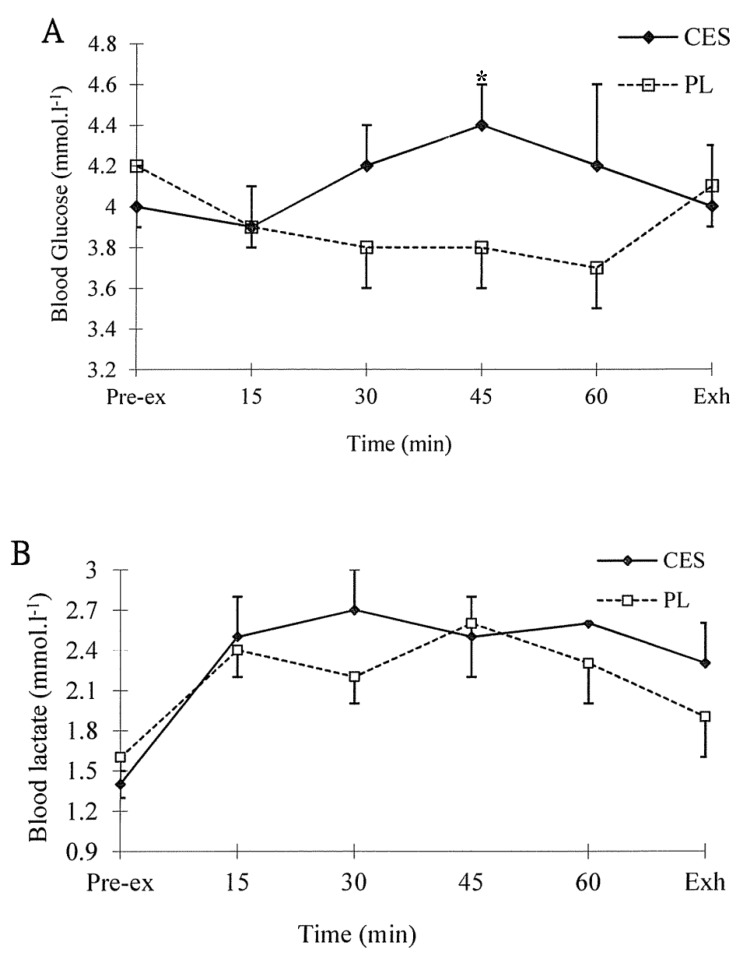
Blood glucose (**A**) and lactate (**B**) concentration during treadmill running. Values (mean ± SEM) are for placebo (PL) and carbohydrate-electrolyte solution (CES) trials. * CES *vs.* PL, *p* < 0.05.

The serum osmolality was similar between the two conditions (CES *vs.* PL: 288.4 ± 2.1 *vs.* 285.9 ± 3.4 mOsm·l^−1^, *p* > 0.05). The blood glucose levels (F = 2.357; *p* = 0.060) and blood lactate concentrations (F = 0.947; *p* = 0.492) were similar between the CES and PL trials (Figure 2A,B). However, at 45min during exercise, the plasma glucose concentration was higher in the CES trial than in PL trial. Furthermore, the plasma glucose concentration increased gradually from the 15-min mark of the CES trial.

### 3.4. Heart Rate, Ratings of Perceived Exertion, Thirst and Abdominal Discomfort

There were no differences in the patterns of change in the exercising HR and perceptual variables between the two trials (Table 1), including RPE (F = 1.767; *p* = 0.129), PTS (F = 1.161; *p* = 0.347), and PAS (F = 0.046; *p* = 0.999).

**Table 1:** Summary of the heart rate, rate of perceived exertion (RPE), perceived thirst scale (PTS) and perceived abdominal discomfort scale (PAS) during running in the carbohydrate-electrolyte solution (CES) and placebo (PL) trials, values are presented as mean ± SEM.

	Trial	Run Time (min)							Significance Level
		Pre-ex	Warm-up	15^th^	30^th^	45^th^	60^th^	EXH	
Heart Rate (beats·min^−1^)	CES	63.9 ± 2.1	115.4 ± 9.5	148.4 ± 3.9	150.0 ± 3.5	150.9 ± 3.4	151.4 ± 3.5	151.3 ± 3.2	F = 2.047
	PL	64.0 ± 3.1	135.9 ± 9.4	148.4 ± 2.9	150.0 ± 3.3	150.5 ± 3.7	152.4 ± 3.5	152.4 ± 4.4	*p* = 0.08
RPE (Borg Scale)	CES	7.1 ± 0.4	7.5 ± 0.4	10.3 ± 0.6	11.3 ± 0.7	13.8 ± 0.6	14.3 ± 1.0	18.5 ± 0.6	F = 1.767
	PL	8.0 ± 0.6	9.4 ± 0.5	10.9 ± 0.7	12.4 ± 0.6	13.3 ± 0.7	15.3 ± 0.8	18.9 ± 0.4	*p* = 0.129
PTS	CES	2.1 ± 0.5		3.5 ± 0.7	3.1 ± 0.6	3.3 ± 0.8	3.9 ± 0.8	4.3 ± 0.9	F = 1.161
	PL	3.0 ± 0.8		4.1 ± 0.8	3.4 ± 0.9	3.4 ± 0.9	4.3 ± 0.9	3.9 ± 1.1	*p* = 0.347
PAS	CES	0.3 ± 0.3	0.6 ± 0.4	1.6 ± 0.6	2.4 ± 0.5	3.3 ± 0.7	3.5 ± 0.7	4.0 ± 0.9	F = 0.046
	PL	0.4 ± 0.3	0.9 ± 0.5	1.5 ± 0.6	2.4 ± 0.7	3.1 ± 0.8	3.4 ± 0.8	4.0 ± 0.3	*p* = 0.999

## 4. Discussion

The main finding of the present study was that the overall endurance time was increased with the ingestion of CES compared with placebo solution in active females.

It has been reported that CHO ingestion during endurance exercise improves exercise performance in male subjects in both exercise to exhaustion and time-trial protocols [6]. Although several studies in females found improved performance after CHO consumption [10,11], not all studies have supported this conclusion [9]. In this specific study, no improvement was found in performance during a 24.2-km time trial when CHOs were consumed during exercise [9]. However, in another study that included 2 h of steady cycling plus a time-trial exercise protocol, the authors found that the ingestion of glucose improved exercise performance compared with the ingestion of a placebo [11]. In the present study, an improvement of approximately 16% was gained in the CES trial compared to the PL trial, which was slightly higher than the figure found in a previous study with a cycling to exhaustion exercise protocol [10]. The different performance measurement protocols may be one factor that contributes to the inconsistent findings, because performance as determined by different methods may vary widely [21] and different physiological responses were found between cycling and running tests [22]. Although it has been argued that exercise to exhaustion protocol has relatively poor test-retest reliability [21], it is still one commonly used method to test the endurance capacity. The improved endurance capacity may come from the increased exogenous CHO consumption during exercise, which could maintain the blood glucose concentration and attenuate the rate of glycogenolysis, thereby sparing muscle glycogen stores and delaying the onset of fatigue [8]. In the present study, although no statistical difference was found in the blood glucose concentrations between the two trials, there was a trend (*p* = 0.06) toward higher blood glucose concentrations in the CES trial than in the PL trial. Furthermore, the blood glucose concentration was higher at 45 min of exercise in the CES trial than in the PL trial (Figure 2). In addition, the CHO oxidation rate during the endurance run did not differ significantly between the two trials (Figure 1). These results indicate that the increased exogenous CHO oxidation may not be the major reason for the improved endurance capacity in the present study. Some other factors, such as an attenuation of central fatigue, may also play a role in the improved endurance capacity [7]. Therefore, one limitation of the present study is that the exact mechanism and/or ideal composition and dosage of CES could not be identified. However, it is obvious that commercially available CES is beneficial to the moderate intensity endurance capacity of active females during the follicular phases of the menstrual cycle.

It has been found that the phase of menstrual cycle does not alter the effects of CHO supplementation on performance [10]. In that study, the decrease in the plasma glucose level was attenuated during both the luteal and follicular phases of the menstrual cycle when CHOs were consumed during exercise. On the other hand, another study [11] found that the substrate metabolism and exercise performance were affected by the different phases of the menstrual cycle. The CHO contribution and performance were greater in the follicular phase than those in the luteal phase. The ingestion of glucose during exercise may minimize these effects. Therefore, the findings of the present study further suggest that CHO consumption may improve endurance capacity during the follicular phase. Some studies have found differences according to gender [23,24] in the metabolic responses of CHO ingested during exercise, whereas others did not [25]. Comparing with male, female may prefer to use more lipids but less CHO during endurance exercise [12]. This may be one reason that no difference was found in CHO oxidation rate between two trials in the present study. More studies are obviously needed to clarify whether the conclusions drawn from male subjects can be generalized to female subjects, especially in consideration of the menstrual cycle.

Another factor that may cause fatigue during endurance exercise is dehydration. Even moderate dehydration has been suggested to have negative effects on endurance performance [2,15]. However, in the present study, the hydration status did not seem to differ between the two trials. The finding was supported by the fact that no difference was found in the serum osmolality between the two trials. The regular ingestion of fluids is strongly recommended during endurance exercise [2]. Most commercially available CES are considered to be appropriate for endurance exercise, but the optimal composition is still unclear [2]. A CES not only supplies energy to spare the body’s limited CHO stores, but also replaces electrolytes that are lost in sweat [26]. The ingestion of sodium-containing solutions during exercise has been found to be more effective in preventing a decrease in the plasma volume than the ingestion of pure water [27]. A high sodium content has also been suggested to make the drinks more effective in rehydrating athletes [28]. Therefore, although the potential effects of electrolytes on endurance capacity could not be excluded in the present study, it seems that the improvement of exercise performance should not be the different hydration level between CES and PL trials.

The HR and blood lactate concentrations were kept constant during the exercise in both trials. This indicated that no dehydration occurred and that the internal loads were similar between the conditions. The RPE increased gradually with both treatments and in the same pattern. In addition, the PTS and PAS of the subjects in the present study remained low and were similar in both trials, which indicate that neither CES nor PL caused abdominal problems during exercise.

Another limitation of the present study is that capillary blood samples were collected to measure the blood glucose and lactate concentrations, which can provide only limited information about the metabolic responses during exercise between the two trials. Further studies are still needed to clarify the potential mechanisms behind the improved running performance in active females who consume a CES during endurance exercise.

## 5. Conclusions

In conclusion, the results of the present study further confirm that CES supplementation improves the moderate intensity endurance capacity of active females during the follicular phases of the menstrual cycle. However, the exogenous CHO oxidation does not seem to explain the improved capacity after CES supplementation.
